# Self-Reported Female Orgasm Following Serial Sacroiliac Joint Injections

**DOI:** 10.7759/cureus.16737

**Published:** 2021-07-29

**Authors:** Nicholas R Storlie, Hamid Abbasi

**Affiliations:** 1 Spine Surgery, Inspired Spine Health, Burnsville, USA; 2 Neurosurgery, Ambulatory Surgical Clinic, Tristate Brain and Spine Institute, Alexandria, USA; 3 Neurosurgery, Inspired Spine Health, Burnsville, USA

**Keywords:** sacroiliac joint, sacroiliac joint injection, sacroiliac joint dysfunction, pudendal nerve, sacroiliac joint fusion, extravasation

## Abstract

Sacroiliac joint (SIJ) dysfunction has been increasingly recognized as the underlying pathology responsible for a significant percentage of cases of chronic lower back pain and radiculopathy. Diagnosis of SIJ dysfunction involves multiple provocation tests followed by serial injections of anesthetic, with significant alleviation of pain indicating that the SIJ is the pain generator. One documented complication of SIJ injections is extravasation of injected material from the SIJ capsule, resulting in unintended symptoms. We report the case of a patient who reported experiencing an orgasm following each of her three diagnostic SIJ injections. We hypothesize that this unusual symptom was caused by extravasation of injected material ventrally to the nearby pudendal nerve, a nerve responsible for sensory innervation of the perineum and a mediator of sexual arousal and orgasm.

## Introduction

Sacroiliac joint (SIJ) dysfunction has been implicated as a causal factor in up to 30% of chronic low back pain, one of the most common and debilitating conditions across the world [1,2]. The SIJs connect the ilia with the lateral aspects of the sacrum bilaterally [3]. They consist of a ventral, auricular-shaped synovial capsule anteriorly bordered by the dorsal aspect of the greater sciatic notch and posteriorly by the interosseous ligament region [4]. The joint is encased by numerous strong ligaments, most prominently the ventral, dorsal, and interosseous ligaments, which limit the movement of the joint to 2-3 mm of movement translationally and < 4° of rotation with significant variation between individuals [5].

Although historically viewed as a non-nociceptive and synarthrotic joint, the SIJ is increasingly acknowledged to be innervated, capable of minor mobility, and capable of causing significant low back pain and radiculopathy [3]. There are several common causes of SIJ dysfunction including trauma, pregnancy, and repeated stress which cause pathological changes to SIJ structures such as the joint capsule, surrounding muscles, and ligaments [6]. Physiological disruptions result in the pathological hypermobility of the joint and aberrant joint mechanics which manifest in low back pain and radiculopathy [4].

The diagnosis of SIJ dysfunction typically consists of examination of clinical findings, a battery of provocation tests, and finally an intra-articular SIJ injection of local anesthetic [7]. Although recognition of pain correlating to SIJ dysfunction and provocation tests are useful for identifying patients with possible SIJ pathologies, these tools alone are not able to rule out pain coming from lower lumbar spinal structures or nearby ligaments and musculature. The use of serial SIJ injections following positive clinical and provocative findings has been shown to be a relatively sensitive and specific diagnostic protocol for diagnosing SIJ dysfunction [8]. Although SIJ joint blocks have been shown to be diagnostically valid, there is a possibility of extravasation of injected material to nearby neural structures which can cause unexpected sensory symptoms. We report the case of a 57-year-old female who experienced orgasm-like sensations following serial SIJ injections.

## Case presentation

The patient was a 57-year-old woman who was initially seen in 2019 for lower back pain with right lower extremity radiculopathy. The patient had previously undergone facetectomy, laminectomy, and L4-5 decompression which were unsuccessful in treating her L4-5 degenerative disc disease with herniated nucleus pulposus and stenosis. Lumbar CT was performed which confirmed previous diagnoses and demonstrated scoliosis with a Cobb angle of 21°. The patient underwent L4-S1 oblique lateral lumbar interbody fusion (OLLIF) two months later. Her pain was improved at six-month follow-up but she reported new and old numbness down the right thigh and pain in the buttock area corresponding to common symptoms of SIJ disease. SIJ provocation tests were positive including Faber test, compression, distraction, and Fortin point tenderness. 

Serial diagnostic right SIJ injections were ordered and the first injection was performed. We administered 3 CC of 0.25% bupivacaine to anesthetize the skin before a 22-gauge spinal needle was entered under fluoroscopic guidance. We injected 1 cc of Iohexol (Omnipaque 240) to confirm the correct placement of needles. A mix of 40 mg triamcinolone acetonide (Kenalog) and 2 cc of 0.25% bupivacaine was injected into the joint. When the patient got off of the injection table after surgery, she experienced numbness, tingling, and warmth from her right labia majora through her vagina extending to half of the left labia majora. This sensation extended to her right upper thigh area near the labia. The patient described the feeling as similar to an orgasm shortly after the injection with residual pleasurable sensation lasting for two hours. She also described the feeling as similar to “pins-and-needles” and noted that the genital arousal was nonsexual. The sensation was rated 10/10 in intensity. The patient did not experience pelvic floor contraction, clitoral engorgement, or increased secretions otherwise associated with female orgasm. The intensity of her sensations was increased with vehicle bumps and vibrations on her ride home. 

The patient received two further diagnostic right SI injections at the second and four weeks after her first injection. Following both of these injections, the patient reported similar symptoms, including numbness, warmth, and tingling extended across the same area of her genitals. Compared to her first injection, she rated the symptoms as less intense at 5.5/10 in intensity. Her symptoms lasted two hours before gradually alleviating.

## Discussion

We report the case of a woman who experienced orgasm-like sensations following each of three diagnostic SIJ injections. Although the utility of SIJ diagnostic injections has been validated in the literature, extravasation of injected material has also been documented [9]. This extravasation can cause unexpected sensory symptoms, with several studies documenting lower extremity numbness due to inferior leakage of anesthesia to the sciatic nerve [10,11].

We hypothesize that the patient’s orgasm-like symptoms were the result of extravasation of injected material to the pudendal nerve, the neural structure which provides both motor and sensory innervation to the perineum [12]. The reported area of paresthesia and genital arousal closely matches the dermatome of the pudendal nerve, and genital afferents deriving from the pudendal nerve have been identified as a mediator of genital arousal and orgasm [13]. Notably, the patient’s symptoms closely match descriptions of persistent genital arousal disorder (PGAD), a disorder often caused by neuralgia of the pudendal nerve [14]. This disorder results in involuntary genital arousal which is involuntary, long lasting, and unrelated to subjective sexual desire. We believe it is likely that the patient’s genital paresthesia is related to a similar dysfunction of the pudendal nerve, due to spatial compression or other dysesthesia related to the extravasation of the anesthetic and steroid.

Due to the unique nature of this report and lack of thorough characterization of the effect of extravasated injectate in the area surrounding the SIJ, the findings we report permit multiple hypotheses that should be investigated in further research. Another possible mechanism we find plausible is that the warmth and orgasm-like sensation the patient experienced is due to bupivacaine-induced dilation of the pudendal artery rather than direct action on the pudendal nerve. The pudendal artery branches from the internal iliac artery and eventually crosses the sacrospinous ligament before joining the pudendal nerve in the pudendal canal and eventually supplying superficial and deep perineal tissue [14]. Clinical concentrations of bupivacaine such as the dose used in our injections have been shown to induce vasodilation in peripheral arteries. It is notable that this effect was shown to last only 40 minutes in peripheral skin, though there are no published findings on the duration of effect in other vascular beds [15]. Vasodilation of aspects of the perineum is another factor linked to cases of PGAD, supporting the idea that vasodilation of this area is another possible cause of the patient’s symptoms [16].

**Figure 1:**
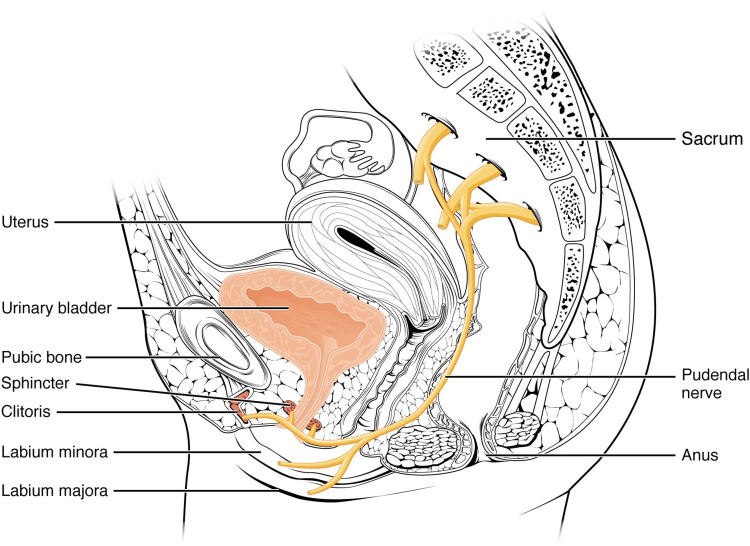
Diagram of the perineal nervous anatomy. OpenStax College, CC BY 3.0 https://creativecommons.org/licenses/by/3.0, via Wikimedia Commons. [18]

The proximity of these structures to previously documented areas of SIJ injection extravasation also indicates their likely role in this incident. The pudendal nerve is formed from contributions of the ventral rami of sacral nerves 2-4, which exit the pelvis through the greater sciatic foramen inferior to the piriformis muscle and bend around the posterior aspect of the sacrospinous ligament before later branching to the innervated tissue [17,12] (Figure 1).

SIJ injection extravasation can be variable, but fluoroscopic studies have observed several more common patterns of contrast medium extravasation from the SIJ capsule [19]. Frequently observed patterns include the leakage of contrast ventrally to the sacral plexus and a trail of contrast leaking inferiorly into the presacral region. These findings demonstrate that extravasations from the SIJ frequently approach areas near the pudendal nerve and pudendal artery, supporting the hypothesis that the patient’s symptoms were caused by incidental application of local anesthesia and steroids to one of these structures. 

## Conclusions

Given the frequency of SIJ injections and close proximity of the pudendal nerve to the SIJ anterior capsule, we hypothesize that post-injection stimulation of the genitals or rectum is more common than has been previously realized. These symptoms would likely be underreported due to their unusual nature that patients may perceive as embarrassing. Our clinic has now set up a system to make reporting of these symptoms more likely as they occur. An important limitation of this case report is a lack of fluoroscopic pictures demonstrating extravasation of the injectate. Because this is the first such case we encountered, our collection of imaging and performance of other workups was limited. We hope to make others aware of the possibility of this complication of SIJ injection in order to further characterize this phenomenon in future occurrences.

The finding that the extravasation of local anesthetic and steroids to the area surrounding the SIJ can cause orgasm-like symptoms also raises questions about the possibility of using a similar technique in order to treat patients suffering from anorgasmia. Further investigation is required as to the specific biological mechanism of stimulation, but pudendal nerve activation appears to be a potential means of causing orgasm-like experiences even in the absence of other stimuli. Anorgasmia is an incredibly common condition among women and men, with up to 43% of American women reporting inability to achieve orgasm and lack of interest in sex. Pudendal nerve stimulation and other methods of sacral neuromodulation are well established means of treating voiding dysfunctions such as neurogenic bladder, but the authors are unaware of the use of pudendal nerve interventions to treat anorgasmia. This could be a promising avenue of research in order to address a common dysfunction.
